# A method of assessing the resilience of whole communities of children: An example from rural Australia

**DOI:** 10.1186/1753-2000-6-17

**Published:** 2012-05-04

**Authors:** Debra A Dunstan, Anna K Todd

**Affiliations:** 1 Discipline of Psychology, School of Behavioural, Cognitive and Social Sciences, University of New England, Armidale NSW 2351, Australia

## Abstract

**Background:**

Children living in socioeconomic disadvantage are at risk of poor mental health outcomes. In order to focus and evaluate population health programs to facilitate children’s resilience, it is important to accurately assess baseline levels of functioning. With this end in mind, the aim of this study was to test the utility of 1) a voluntary random sampling method and 2) quantitative measures of adaptation (with national normative data) for assessing the resilience of children in an identified community.

**Method:**

This cross-sectional study utilized a sample of participants (*N* = 309), including parents (*n* = 169), teachers (*n* = 20) and children (*n* = 170; age range = 5-16 years), recruited from the schools in Tenterfield; a socioeconomically disadvantaged community in New South Wales, Australia. The Strengths and Difficulties Questionnaire (SDQ; including parent, teacher and youth versions) was used to measure psychological well-being and pro-social functioning, and NAPLAN results (individual children’s and whole school’s performance in literacy and numeracy) were used to measure level of academic achievement.

**Results:**

The community’s disadvantage was evident in the whole school NAPLAN performance but not in the sample’s NAPLAN or SDQ results. The teacher SDQ ratings appeared to be more reliable than parent’s ratings. The voluntary random sampling method (requiring parental consent) led to sampling bias.

**Conclusions:**

The key indicators of resilience - psychological well-being, pro-social functioning and academic achievement – can be measured in whole communities using the teacher version of the SDQ and whole school results on a national test of literacy and numeracy (e.g., Australia’s NAPLAN). A voluntary random sample (dependent upon parental consent) appears to have limited value due to the likelihood of sampling bias.

## Background

Ecological systems theory [1,2] posits that children’s developmental outcomes are the result of the outworking of a series of reciprocal interactions between the child’s biological and personal characteristics, and influences from the family, school and wider community [3-7]. In Australia, a substantial body of evidence indicates that people residing in agriculture-based rural communities have poorer mental health than their urban counterparts [8-10]. This is seen as secondary to higher rates of unemployment [11], lower levels of income [12], and limited social inclusion [13]. The environmental antecedents of these circumstances include: climate change, prolonged drought and rural restructuring [14]; restricted socio-cultural opportunities [15]; and, limited access to health care services [16]. In line with ecological theory, data has emerged showing the negative impact of these influences on children’s current and future functioning [17]; consequently, calls to protect rural children’s mental well-being - via school-based interventions to build resilience [18]- are on the rise [19].

In order to focus and evaluate population health resilience programs, the level of children’s current resilience – defined in this study as ‘manifest competence in the context of significant challenges to adaptation or development’ [6] (p. 206) – needs to be assessed prior to the commencement of intervention activities [20]. Scales have been developed to measure adults’ resilience, but of the six psychometrically-sound scales available, only one has been identified as suitable for use with minors, and only then with adolescents [21]. Consequently, in experimental studies of resilience in children, the construct is usually operationalized as adaptive functioning, demonstrated in the outcomes of mental wellbeing, social competence, and academic achievement [3,5,22].

At the time of conducting this study, there was a dearth of literature reporting on the measurement of resilience in whole communities of children; particularly those living in rural Australia [7]. Therefore, as part of a multidisciplinary pilot project to trial methodologies and gather background information for a large-scale study [23], this study aimed to examine the utility of using 1) a voluntary random sample; and, 2) quantitative measures of adaptation (with Australian national norms), for assessing the dimensions of resilience in a community of children.

## Method

### Participants

The total sample (*N* = 309), including parents (*n* = 169), teachers (*n* = 20) and children (*n* = 170; age range = 5-16 years; 43% male; 57% female) were recruited from all three schools in the town of Tenterfield, New South Wales (NSW) Australia. The number and gender of child participants by age is shown in Table 1, and the percentage of children per school year/age group is shown in Table 2 (Note: this latter categorization was used to permit the comparison of scores on the quantitative measures with national norms that are reported in school year/age range categories only). The total sample of children comprised the following groups: 33 students (39.4% male and 60.6% female) from Tenterfield High School (Government school) where the response rate was approximately 14%; 102 students (40.2% male and 59.8% female) from the Sir Henry Parkes Memorial Public School (Government school) where the response rate was 43.4%; and, 35 students (54.3% male and 45.7% female) students from the St Joseph’s Primary School (Non-government Catholic school) where the response rate was 21.2%. The overall response rate was 27%.

**Table 1:** *Frequency of Male and Female Child Participants by Age (n = 169)*

**Age in years**	**Sex**	
	**Male**	**Female**
5	5	10
6	8	13
7	11	10
8	6	11
9	11	13
10	6	12
11	7	4
12	7	5
13	4	9
14	2	7
15	4	2
16	2	0

**Table 2:** *Frequency and Percentage of Participants per School Year/Age group*

**School Year Age range (in years)**	**Frequency (*N*)**	**Percentage of Total Sample**
Year K/1/2 (5–7)	65	38.2
Year 3/4 (8–9)	36	21.2
Year 5/6 (10–12)	35	20.6
Year 7/8 (13–14)	22	12.9
Year 9/10 (15–16)	11	6.5
Missing	1	0.6
Totals	170	100

### Measures

Resilience can vary across domains of functioning [24]; therefore, a range of indicators of competency - psychological well-being, pro-social functioning and academic achievement – were measured with tools having extensive national normative data.

#### Psychological well-being and pro-social functioning

##### *Strengths and Difficulties Questionnaire (SDQ)*

This 25-item screening instrument for child and adolescent mental health problems [25] is comprised of five scales - Emotional Symptoms, Conduct Problems, Hyperactivity/Inattention, Peer Relationship Problems, and Pro-social Behavior – which are designed to detect emotional and behavioral difficulties and social functioning. The questionnaire is for use with 4 to 17 year olds and has parent, teacher and youth self-report versions. Scale scores can range from 0 to 10. A Total Difficulties score is generated by summing the scores from all the scales (except the Pro-social Behavior scale) and can range from 0 to 40. Total scores can be classified in band-levels of functioning - “normal”, “borderline”, or “abnormal” - and Australian [26] as well as international norms are available (http://www.sdqinfo.org/g0.html). The SDQ has been shown to have satisfactory psychometric properties: in this study, the mean Cronbach’s alpha was .70 (Total Difficulties Score: alpha = .74; Pro-social Behavior: alpha = .69) and the mean cross-informant correlation was *r* = .36. This SDQ is currently being used as a measure of emotional, behavioral and social functioning in the “Growing up in Australia: Longitudinal Study of Australian Children (LSAC)”; a study which commenced in 2004 and has been following the development of 10,000 children and families from all parts of Australia [27].

#### Academic achievement

##### *National Assessment Program—Literacy and Numeracy (NAPLAN)*

Since May 2008, the Australian government has required all students to sit a NAPLAN test [28] (on the same day nation-wide) in Years 3, 5, 7 and 9. Testing is undertaken at the students’ school. Results are reported on individual scales for the assessment domains of Reading, Writing, Spelling, Grammar and Punctuation, and Numeracy. Each scale has 10 level-of-achievement bands which represent increasing complexity of skills and understanding. Six of the 10 bands, overlapped across adjacent Years, are used for reporting students’ performance in each Year level: Year 3, Bands 1 – 6; Year 5, Bands 3 – 8; Year 7, Bands 4 – 9; and, Year 9, Bands 5 – 10. The second bottom band at each Year level represents the National Minimum Standard for that level. Scaled scores allow comparisons to be made with national averages.

### Procedure

#### The pilot community

The rural town of Tenterfield (resident population = 3,129 people; children aged 5 to14 years = 1,020) was chosen for this cross-sectional study for two reasons. First, the town is affected by adversity, thus creating the necessary requirement for the demonstration of resilience. Specifically, at the time of this study the community had been impacted by prolonged drought and changes in socioeconomic status [29] resulting in a ranking at the 2^nd^ decile on the Index of Relative Socio-Economic Advantage and Disadvantage (an Index derived from Census variables such as low income, low educational attainment, unemployment, and dwellings without motor vehicles) and the 3^rd^ decile on the Index of Economic Resources (derived from Census variables like residents’ incomes, housing and assets) [30]. Second, the town is located within the same geographical region as the researchers’ institution which had practical benefits for the conduct of the study.

#### *The use of a voluntary random sampling method*

The recruitment of a voluntary random sample - from which generalizations about the population of interest are made - is a common procedure in quantitative research methodology [31]. In this study, participants were recruited via the Tenterfield schools (one high school and two primary schools) following receipt of ethics approval from the University of New England Human Research Ethics Committee (HREC), the State Education Research Approval Process (SERAP) and the Catholic School Office (CSO). The approval process, from the initial writing of applications to final approval by all three institutions, took just under seven months.

The Principal was the key contact person at all schools and took responsibility for inviting students, parents, and associated classroom teachers, to participate in the study. All students in both primary schools, and students up to the age of 16 years at the high school, were included. As the study was conducted in November, the Year 12 students (18 year olds) had graduated, and the Year 11 students (17 year olds) had commenced Year 12 studies. Therefore, the high school Principal recommended that participation be restricted to students in Year 10 (16 year olds) and below, which also coincided with the age-ranges in which NAPLAN results are available. Classroom teachers issued all students (in the 5 to 16 years age range) with information sheets, a parent version of the SDQ, and parental consent forms (which sought information about the child’s age, sex, and school Year, and requested consent for (a) completion of the SDQ by the classroom teacher, and, (b) access to the child’s NAPLAN results). The teacher’s role was limited to distributing the research packages, collecting the returned forms, and completing an SDQ for participating children.

In accordance with Ethics Committee requirements and the concept of a “mature minor” [32], research packages for high school students (aged 13 to 16 years) also included an assent form and a youth self-report version of the SDQ. Students who returned the signed parental consent form and - where appropriate - the assent form, participated in the study. Without the researcher’s knowledge, the Sir Henry Parkes Memorial Public School facilitated participation by offering a small incentive to the children (a chocolate frog) for the return of the paperwork. The response rate at this school (43.4%) was more than double that of the other schools. For comparative purposes, each school’s total NAPLAN results were accessed from the Australian Government’s *My School* website (http://www.myschool.edu.au/).

## Results

The findings provided information about the utility of a voluntary sample, and measurement tools with national norms, for assessing adaptation in whole communities of children.

### Psychological well-being and pro-social functioning

The means, standard deviations, score ranges and classifications for the outcome measures of psychological well-being (SDQ Total Difficulties score) and pro-social functioning (SDQ Pro-social Behavior score) are displayed in Table 3. The data shows that the mean scores on both SDQ scales are within the ranges considered by Mellor [26] to be “normal” (see http://www.sdqinfo.org), nevertheless the scores for the Total Difficulties scale extended across the “normal”, “borderline” and “abnormal” classifications, as defined in the scoring instructions. While parents’ classifications varied significantly from the distribution in Australian norms – specifically, fewer children were categorized in the “normal” range and more in the “borderline” range - teacher and youth self-report ratings of these same children produced classifications that were not different to the norms.

**Table 3:** *Means, Standard Deviations, Ranges and Australian Norms for Pros-social Functioning (SDQ Pro-social Behavior Scores) and Psychological Well-being (SDQ Total Difficulties Scores) and Number, Percent and Category Classifications for Psychological Well- being and Comparisons with Australian Norms, by Informant*

**Informant**	**Pro-social Functioning (SDQ Pro-social Behavior scores)**^**1**^			**Psychological Well-being (SDQ Total Difficulties Scores)**^**2**^				
	**Continuous score data (Possible score range: 0–10)**			**Continuous score data (Possible score range: 0–40)**		**Categorical data**		
	**Sample *M* (SD) Range**	**Australian norms**^**3**^** M (SD)**	**Sample *M* (SD) Range**	**Australian norms**^**3**^** M (SD)**	**Category Classification**	**Sample *N* (%) in category**	**Australian norms**^**3**^**(% in category)**	**Difference in category ratings**
Parent (*n* = 168)	8.2 (1.7) (2–10)	8.3 (1.7)	9.8 (6.8) (0 – 27)	8.2 (6.1)	Normal: Borderline: Abnormal:	118 (70.2) 22 (13.1) 28 (16.7)	82.0 6.0 12.0	χ^2^ (2) = 19.97, *p* < .001
Teacher (*n* = 154)	7.6 (2.3) (1–10)	7.8 (2.1)	6.6 (6.3) (0–26)	6.5 (6.0)	Normal: Borderline: Abnormal:	123 (79.9) 16 (10.4) 15 (9.7)	76.9 6.0 8.5	χ^2^ (2) = 2.85, *p* = .313
Student (*n* = 30)	7.6 (1.8) (3 – 10)	8.0 (1.7)	10.7 (6.0) (2 – 23)	9.0 (5.6)	Normal: Borderline: Abnormal:	23 (76.7) 4 (13.3) 3 (10.0)	86.0 8.2 5.8	χ^2^ (2) = 2.18, *p* = .336

^1^Normal scores range = 6 – 10

^2^Normal scores range = 0 – 13

^3^http://www.sdqinfo.org/norms/AusNorm1.pdf.

### Academic achievement

Table 4 displays the NAPLAN results for the child participants (Note: data is provided in Band levels only). As NAPLAN testing occurs bi-annually for individual students, and does not commence until Year 3, scores were available for only 34% (*n* = 53) of the sample in the year this study was conducted. Table 4 shows the percentage of these students who were performing at their school average, or above, on each domain. These results indicate that the sample is biased towards the stronger performers in each school: St Joseph’s Primary School - 90% performing at or above the school average on all domains; Tenterfield High School - an average 84% performing at or above the school averages; and, Sir Henry Parkes Memorial Public School – an average 74% performing above the school’s averages. Thus, although the use of an incentive at Sir Henry Parkes School drew a sample in which 25% of participants were performing below the school’s average (compared to only 10% of the St Joseph’s sample), the total sample was non-representative of the population of Tenterfield school children in terms of academic achievement. This was significant because above average intelligence/academic achievement is a protective factor strongly influencing resilience [33]. Therefore, for further analyses, whole school NAPLAN results were obtained for the study year - 2009 - by accessing the Australian Government’s *My School* website (http://www.myschool.edu.au/) and comparing these findings with national NAPLAN results (http://www.naplan.edu.au/).

**Table 4:** *NAPLAN Results per School Showing Percentage of Child Participants Performing at or Above the School Averages (n = 53)*

**NAPLAN domain**	**% Performing at School average or above**		
	**Tenterfield High** **(*n* = 14)**	**Sir Henry Parkes** **(*n* = 29)**	**St Joseph’s** **(*n* = 10)**
Reading	92.8	69.0	100.0
Writing	85.7	79.3	90.0
Spelling	85.7	72.4	80.0
Grammar and Punctuation	78.6	75.9	90.0
Numeracy	78.5	72.4	90.0

Table 5 displays the percent of all Years 3, 5, 7 and 9 students in Tenterfield who performed at or above the National Minimum Standard in Reading, Writing, Spelling, Grammar and Punctuation, and Numeracy, by school. The results in Table 5 indicate that the non-Government school students (i.e., those at St Joseph’s Primary School) were performing at a level either approaching, or comparable to, national minimum standards on all domains. In contrast, the performance of students at the Government schools – Tenterfield High and Sir Henry Parkes Memorial Public School - varied across the domains and, with the exception of Numeracy in Years 5 and 9, scores fell well below national minimum requirements. An even clearer picture emerged when performance was compared to national averages (see Table 6).

**Table 5:** *Percent of All Years 3, 5, 7 and 9 Students Performing at or above the National Minimum Standard in Reading, Writing, Spelling , Grammar and Punctuation, and Numeracy by School in 2009 (National percentages in brackets)*

**School**	**Year**	**Domain**				
		**Reading** **(National)**	**Writing** **(National)**	**Spelling** **(National)**	**Grammar &Punctuation (National)**	**Numeracy** **(National)**
Tenterfield High School	7	76 (93.9)	63 (92.5)	70 (92.9)	65 (92.0)	73 (94.8)
	9	69 (92.2)	43 (87.7)	55 (89.7)	66 (90.3)	90 (94.9)
Sir Henry Parkes Memorial Public School	3	88 (93.8)	74 (95.6)	75 (92.2)	58 (92.5)	73 (94.0)
	5	63 (91.7)	71 (92.8)	79 (92.4)	68 (92.0)	90 (94.2)
St Josephs Primary School	3	93 (93.8)	90 (95.6)	91 (92.2)	88 (92.5)	91 (94.0)
	5	86 (91.7)	89 (92.8)	85 (92.4)	79 (92.0)	89 (94.2)

**Table 6:** ***Percentage of Sample and Whole Year Classes – by School - Performing at or above the Australian National Average***^***1b***^

	**Tenterfield High School**		**Sir Henry Parkes Memorial Public School**		**St Joseph’s Primary School**	
**NAPLAN domain**	**Study sample** **(*n* = 14)**	**Years 7 & 9**	**Study sample** **(*n* = 29)**	**Years 3 & 5**	**Study sample** **(*n* = 10)**	**Years 3 & 5**
Reading	71.4	Yr 7–35 Yr 9 - 53	62.0	Yr 3–46 Yr 5 - 24	70.0	Yr 3–59 Yr 5 - 59
Writing	51.7	Yr 7–23 Yr 9 - 44	72.4	Yr 3–51 Yr 5 - 38	80.0	Yr 3–64 Yr 5 - 62
Spelling	64.3	Yr 7–33 Yr.9 -34	75.8	Yr 3–58 Yr 5 - 42	50.0	Yr 3–61 Yr 5 – 50
Grammar & Punctuation	64.3	Yr 7–31 Yr 9 -22	72.4	Yr 3–55 Yr 5 - 42	70.0	Yr 3–78 Yr 5 - 62
Numeracy	57.1	Yr 7–29 Yr 9 - 33	51.7	Yr 3–44 Yr 5 - 16	60.0	Yr 3–95 Yr 5 - 71

^1^Calculations are based on the percent performing in the relevant Bands.

Table 6 displays the percentage of participants, and whole class groups – by school – performing at the national average, or above, in each NAPLAN domain. The data in this table show that, at a minimum, 50% of the study sample was performing at this level or above; however, when the results of whole class groups were reviewed, the findings were less favorable. On average, only 33% of the high school students, and 42% of the government primary school students, were performing at or above national averages. In contrast, 66% of the non-government primary school students were performing at this level.

## Discussion

The findings and procedural experiences from this study form the basis of recommendations for the assessment of the resilience of children living in identified communities. First, we found that it takes considerable time to set up a study involving child participants. Scoping and liaison is necessary to determine that adversity is present in a community and to secure the engagement of key parties. Many months may be required to complete these activities and obtain ethics approval from multiple stakeholders. Second, our findings showed that a voluntary random sampling method (requiring parental consent) led to sampling bias. Third, we found that measurement tools with national normative data can be used to assess adaptation in key areas of functioning. These latter points are discussed below.

### The use of a voluntary random sampling method

This study’s recruited sample was found to be biased towards students with better academic achievement, and thus it was not representative of all children (5 to 16 years of age) living in Tenterfield NSW. While this bias was reduced in one school - which achieved a response rate of 43% by offering a small incentive - the sample nevertheless remained atypical of that school. In line with the findings of other research where parental consent for participation was required [34], our results suggest that the recruitment of a large sample, from which reliable generalizations can be made, is unlikely to occur using this procedure. This opinion is also supported by information obtained from a focus group conducted by our multidisciplinary collaborators [35] wherein it was revealed that the parental response rate to school surveys is typically “less than 20%” and “only (from) the best functioning families” (p. 7). Thus, we conclude that a volunteer random sample is unlikely to be representative of all children in a community suffering from adversity; therefore, other means are required to capture a true assessment of overall resilience and to identify the specific risk factors impacting on more vulnerable sub-groups. We suggest that the most appropriate way to assess the resilience of whole communities of school-aged children is to use measures and procedures that provide population data. These are described below.

### The use of quantitative measures of resilient adaptation

The SDQ was used to measure “psychological well-being” and “pro-social functioning”. Results showed concordance between all raters for pro-social functioning (the mean scores were all in the “normal” range). However, while the teacher and youth self-report classifications of psychological well-being were consistent with average population ratings, the parent’s ratings of these same children were not. Parents rated their children as having greater pathology than perceived by the other raters: specifically, they rated only 70% of the children in the “normal” category, whereas the normative data suggests 82% would be in this group. Given the discrepancy between the rater’s findings, the reliability of the parents’ ratings is questioned. While we have no information about the parents’ functioning, based on the level of community disadvantage it may be possible – as found by other researchers - that parental stress [36], or compromised parental mental health [37], led to the perception of greater emotional and behavioral disturbance in the children than was identified by the other raters. On the other hand, there is nothing in our findings to cast doubt on the reliability of the teacher’s ratings and, as it would be possible to gain whole class scores in this way, we conclude that (Ethics Committee approval permitting) teacher evaluations of whole classes appears to be a feasible way of measuring psychological well-being and pro-social functioning in communities of children. That said; we do not suggest that parents’ perspectives be ignored. Indeed, as children’s behavior is significantly influenced by context [38], parents’ evaluations could potentially contribute important and valuable information for intervention planning.

This study has also shown that by accessing publicly-available literacy and numeracy results (for example, NAPLAN data in Australia) the outcome - “academic achievement” – can be easily ascertained for whole communities of children. As noted above, our sample’s NAPLAN results indicated that it was not representative of the population of interest (i.e., the majority of participants were performing above their school’s averages). Not surprisingly, when whole school performances were obtained the impact of community socioeconomic disadvantage – not evident in our recruited sample - became clear, along with the academic areas requiring remediation. Therefore, again we suggest that this epidemiological approach be taken if a true picture of overall community functioning is to be gained.

### Limitations

As noted throughout this section, recruitment of a voluntary sample of participants led to sampling bias and as such we did not appear to recruit the children most at risk of poor adaptation. However, whilst we suggest that more accurate results will be obtained using population data, we have not fully tested this recommendation. Similarly, we acknowledge that our conclusions are based on pilot work only.

Another limitation is an ethical matter. While our methods met the requirements of the approving Ethics Committee and our professional Code of Ethics, we wish to acknowledge that a higher standard would have involved obtaining written assent from all child participants, not just those 13 years and older. These limitations aside, we offer the following conclusions.

## Conclusions

It is important to assess the functioning of children exposed to adversity if interventions to protect their mental well-being are to be focused and the outcomes suitably evaluated. This study found that it is feasible to measure the key indicators of adaptation within identified communities using measures (with national normative data) that are available in the public domain. Psychological well-being, pro-social functioning and academic achievement can be measured using the teacher version of the SDQ and a national literacy and numeracy performance data base (i.e., NAPLAN in Australia). Ethics Committee approval pending, it may be possible to obtain anonymous results on these measures without the need for parental consent. Such a strategy would potentially lead to the recruitment of a large and unbiased sample, unlike that of the voluntary random sample (dependent upon parental consent) used in this study. Lastly, future researchers might consider supplementing this data with qualitative findings from a purposive subsample from the chosen community’s most disadvantaged children. This procedure would give children “a voice” to express their perspectives [39] and identify the specific risk factors impacting upon them.

## Competing interests

The authors declare that they have no competing interests.

## Author’s contributions

DD = 50%, AT = 50%. AT conducted the literature review, prepared the ethics applications, assisted with data collection and analysis, and helped draft the manuscript. DD planned the study, assisted with data collection and analysis, and drafted the first version of the manuscript. Both authors read and approved the final version of the manuscript.
